# Detection of autoantibodies against Interferon Type I in patients with autochthonous West Nile Virus encephalitis

**DOI:** 10.1016/j.bbih.2025.101085

**Published:** 2025-07-30

**Authors:** Jan Braune, Lorenz Pechstein, Christian Meisel, Tim Meyer, Julia Melchert, Victor Max Corman, Christiana Franke, Thomas Schneider

**Affiliations:** a Department of Medicine III (Hematology, Oncology and Tumorimmunology), Charité – Universitätsmedizin Berlin, Hindenburgdamm 30, 12203, Berlin, Germany; b Department of Neurology, Charité – Universitätsmedizin Berlin, Germany; c Institute of Medical Immunology, Charité – Universitätsmedizin Berlin, Hindenburgdamm 30, 12203, Berlin, Germany; d Labor Berlin – Charité Vivantes GmbH, Sylter Strasse 2, 13353, Berlin, Germany; e Institute of Virology, Charité – Universitätsmedizin Berlin, Germany and German Centre for Infection Research (DZIF), Associated Partner Site Charité, Charitéplatz 1, 10117, Berlin, Germany; f Department of Medicine I (Gastroenterology, Rheumatology, Infectious Diseases), Charité – Universitätsmedizin Berlin, Hindenburgdamm 30, 12203, Berlin, Germany

**Keywords:** West Nile, Encephalitis, Autoimmunpathology, Neurological symptoms, Intrathecal viral Replication, Germany

## Abstract

West Nile virus (WNV) is a neurotropic flavivirus that has become a global concern because of its rapid geographical spread and its capacity to cause severe disease in humans. Although often asymptomatic, infection can lead to life-threatening neuroinvasive disease (WNND) in a small percentage of cases, particularly among elderly individuals and individuals with compromised immune responses. We present three cases of autochthonous WNND from Germany from 2023 to 2024, which showcase various neurological manifestations and their correlation with immune responses. Two elderly men developed severe encephalitis symptoms, whereas one patient presented with only mild encephalitis symptoms but severe flaccid paresis. Notably, two patients (Patients One and Two) had neutralizing autoantibodies against interferon-α2 and interferon-ω, along with neutralizing antibodies against interferon-β in Patient One. This may implicate impaired viral immunity and severe disease progression. Complete genome sequencing revealed lineage 2 sequences with high similarity to WNV sequences obtained from other patients, birds, and mosquitoes in Germany. Our report raises concerns about the underdiagnosis of WNV in Germany, where human cases remain underreported despite the presence of endemic infections in animals and competent mosquito vectors. Given the increasing prevalence of WNV in Europe, intensified surveillance and awareness are critical. The variability in clinical presentations and the potential for fatal outcomes underscore the importance of early recognition and individualized management strategies.

## Highlights

• IFN-α/ω autoantibodies linked to severe West Nile encephalitis.
• Elderly patients with IFN autoantibodies face severe neurological outcomes.
• WNV lineage 2 strains circulate in humans, birds and mosquitos in Germany.
• Underreported WNV in Germany calls for intensified surveillance.

## 1 Introduction

West Nile virus (WNV) is a mosquito-borne, neurotropic flavivirus capable of triggering life-threatening disease in humans. In recent years, WNV infections have been reported in at least 60 countries across all continents, and the virus continues to spread to new regions (Chowdhury and Khan, 2021). It is now recognized as one of the leading mosquito-borne diseases worldwide. Nevertheless, only a few cases have been described in Germany to date, and clinical characterization of severe cases is lacking. Surveillance data suggest that WNV is endemic among animals in certain regions of Germany; however, human cases are rarely reported (Robert-Koch-Institut. Survstat; Friedrich- Loeffler et al.). This may be due to the predominantly asymptomatic nature of human infections. Nonetheless, a small proportion of individuals infected with WNV develop life-threatening disease characterized by prominent neurological symptoms (Petersen et al., 2013). These disease courses may be fatal and often result in long-term neurological disabilities. The reasons for these variable outcomes are not yet fully understood. Recent publications have suggested that circulating antibodies against interferon-α2 and interferon-⍵ may increase the risk of severe infection, with the prevalence of these antibodies increasing with age (Gervais et al., 2023).

We describe three life-threatening cases of autochthonous WNV infection that share some common general symptoms but exhibit distinct neurological manifestations, which appear to be associated with differences in the immune response and virological presence in the central nervous system. Two elderly men developed severe encephalitis symptoms accompanied by increased inflammation in the cerebrospinal fluid (CSF), whereas the female patient primarily presented with flaccid paresis, reminiscent of inflammatory demyelinating polyradiculoneuropathies. We demonstrated circulating autoantibodies against interferon in the blood which may explain their divergent clinical presentations. Furthermore, by reviewing human and veterinary epidemiological data, we raise the question of whether the true prevalence of WNV in Germany might be underestimated.

## 2 Materials and methods

### 2.1 Detection of autoantibodies against interferons

IFN-AABs were detected via an electrochemiluminescence immunoassay (ECLIA) platform (MSD, Rockville, USA), as described recently (Meisel et al., 2021). Briefly, MSD GOLD 96-well small spot streptavidin screen plates (MSDs) were washed with wash buffer (MSD) and blocked with 150 μl of blocking buffer (Thermo Fisher, Waltham, USA) per well at 4 °C overnight. All further incubations were performed for 60 min at room temperature. After blocking, the plates were incubated with IFN-α2 (Merck Sharp & Dohme, Kenilworth, USA), IFN-⍵ (Peprotech, Rocky Hill, USA), IFN-β (Bayer Vital GmbH) or IFN-γ (Imukin, Boehringer Ingelheim, Berlin Germany) linked to biotin (Thermo Scientific, Waltham, USA). Next, the plates were incubated with patient serum. Unless otherwise stated, the serum was diluted 1:100 in blocking buffer. Cytokine AABs were detected via the use of a monoclonal mouse antibody against human IgG (D20JL-6, MSD). After incubation and washing, 150 μl of read buffer (ReadBufferT (4x), MSD) was added, the mixture was incubated for 10 min at room temperature, and the plates were analyzed via a MESO QuickPlex SQ 120 analyzer (MSD). The data are shown as light signal counts (LSC). Samples were considered positive when the respective LSC value exceeded the 97.5th percentile of the AAB levels of the analyzed sera from 667 healthy controls (cutoff for IFN-ɑ = 1980 LSC, IFN-⍵ = 1961 LSC, and IFN−γ=1516LSC) (Akbil et al., 2021).

### 2.2 Competition assays

The specificity of the IFN-α2-AABs and IFN-⍵-AABs was assessed via a competition assay using unbiotinylated IFN-α2 or IFN-⍵. The sera of interest were diluted 1:100 with blocking buffer and incubated overnight at 4 °C with 2.5 mg/ml, 0.025 mg/ml, or 0.00025 mg/ml unbiotinylated IFN-α2 or IFN-⍵. After incubation, reverse ELISA was performed as described above. IFN-AABs in a given serum sample were scored specifically when preincubated with the highest concentration of unlabeled IFN-α2 or IFN-⍵, which resulted in at least a fourfold reduction in the number of LSCs in comparison to that in the same serum sample without IFN-α2 or IFN-⍵ preincubation.

### 2.3 Phosphorylation of STAT1

One hundred microliters of heparinized whole blood from healthy controls and patients was stimulated for 13 min at 37 °C with 1, 10, 100 or 1000 ng/ml recombinant human IFN-α2 (Merck Sharp & Dohme), recombinant human IFN-γ (Boehringer Ingelheim) or recombinant human IFN- β (Bayer Vital GmbH). After 8 min of stimulation, a mixture of surface antibodies (CD16-PE, CD56-PE, CD19-PC5.5, CD3-AF750, CD14-ECD, CD4-PacBlue, and CD45-KrO; Beckman Coulter) was added. After 5 additional minutes, 50 μl of PerFix EXPOSE buffer 1 (Beckman Coulter) was added for 10 min at room temperature, followed by the addition of 1 ml of PerFix EXPOSE buffer 2 for 5 min at 37 °C for cell fixation and permeabilization. The cells were stained with AF488-conjugated antibodies against pSTAT1 (pY701, clone 58D6; Cell Signaling Technology) for 30 min at room temperature in the dark. The samples were washed twice, and a 10-color NaviosEX flow cytometer (Beckman Coulter) was used. Navios software was used for data analysis (Chowdhury and Khan, 2021). The data are shown as the mean fluorescent intensity (MFI) signal of phospho-STAT1 in CD45+CD14^+^ monocytes.

### 2.4 Complete genome sequencing of WNV strains

Sequencing was performed as described earlier (Ruscher et al., 2023). Viral sequence data are available under the GenBank accession numbers PQ328936, PV221468, and PV221469. Reference sequences obtained from GenBank include their accession number, country of origin, sampling year and host name.

## 3 Results

The clinical courses of the three patients who presented at our clinic between 2023 and 2024 are detailed below. All patients required several days to weeks of intensive care unit treatment. For each case, we first describe the clinical presentation and course, then the virological findings, and finally the auto-antibody analyses. For detailed clinical and diagnostic findings see Table 1.

**Table 1:** Summary of the demographics, clinical features, laboratory investigations, neuroimaging findings, and treatment of the three cases.

	Patient #1	Patient #2	Patient #3
**Demographics**			
**Gender**	male	male	female
**Age**	80	86	69
**Comorbidity**	none	Giant cell arteritis Arterial hypertension	Rheumatoidic arthritis
**Clinical Presentation**			
**Days from Symptoms to hospital admission**	4	4	10
**Starting Symptoms**	Upper respiratory symptoms, diarrhea, fever	Fever, diarrhea	Vomiting, diarrhea
**Symptoms at admission**	Fever, maculopapula rash, muscle weakness, aphasia	Fever, confusion, tremor, muscle weakness	Psychomotor retardation, tetraparesis and dyarthria
**Neurological examination**	Cerebellar syndrome with reduced vigilance and gaze-evoked nystagmus and spontaneous nystagmus, visual impairment, slight facial asymmetry on the left, subtle ataxia of the right arm and dysarthria	Fluctuating vigilance, disorientation (deficits in recalling temporal, spatial, and situational information) and pronounced hypokinetic-rigid symptoms and arrhythmic myoclonus in the left arm	Slight cognitive impairment, dysarthria and slight expressive aphasia, mild tetraparesis with generalized areflexia
**Laboratory Parameters**			
Macroscopic characteristics	cloudy	cloudy	clear
WBC count (μl) Reference 0-4	1475	804	50
WBC subpopulations	84 % neutrophiles	67 % neutrophiles	45 % neutrophiles
RBC count (μl) Reference <100	<100	9000	<100
Proteins (mg/l) Reference 150-450	2042	1574	1294
Glucose (mg/dl) Reference 40-70	56	66	59
Lactate (mg/dl) Reference 10-22	29.9	36.9	33.6
Diagnostic PCR Urine	positive	positive	positive
Diagnostic PCR CSF	positive	positive	negative
Diagnostic PCR Blood	positive	positive	positive
IgM Antibodies Serum	positive	positive	positive
IgM Antibodies CSF	negative	negative	negative
Anti-Cytokine Antibodies in Serum	positive	positive	negative
**EEG findings**	Delta-theta mixed activity without evidence of epileptiform potentials	Theta activity with delta components with at least moderate general changes and intermittent right fronto-centro-temporal dysfunction with embedded and underlying 1–2/s delta waves	Right temporo-parietal polymorphic, non-rhythmic theta and delta waves, with occasional spread to the contralateral hemisphere
**MRI findings at admission**	Unspecific T2w hyperintense white matter lesions	unremarkable	Chronic infarct in the right frontobasal region of the anterior cerebral artery (ACA) and early signs of leukoaraiosis on FLAIR sequences
**Electroneurography**	Mixed axonal-demyelinating polyneuropathy with mainly motor deficit and asymmetric involvement of the legs	Not performed	Predominantly axonal sensorimotor polyneuropathy
**Electromyography**	Moderate pathological spontaneous activity (PSA) with positive sharp waves (PSW) and fibrillation potentials, reduced interference pattern with decreased recruitment during maximal voluntary innervation	Not performed	Mild chronic neurogenic alterations, including minimal pathological spontaneous activity (PSA) and reduced recruitment
**Management**	Corticosteroids, IVIGs	Corticosteroids	Corticosteroids, IVIGs
**Long term outcome**	Gait insecurity, fatigue, cognitive deficits	Walking with a walker-rollator is limited to a maximum distance of 20 m, Fatigue	Muscle weakness, mild paraparesis, gait insecurity, hypesthesia of the legs, use of walker-rollator necessary
**Barthel Index (long-term)**	95 points	25 points	85 points

### 3.1 Clinical data

An 80-year-old, sprightly active male psychotherapist with no significant medical or travel history developed fever, upper respiratory symptoms, and diarrhea after a boat trip in Berlin. Antibiotic treatment with amoxicillin was initiated by his general practitioner. On day four, his fever worsened, a maculopapular rash appeared, and weakness developed, leading to hospital admission. At admission, he exhibited moderate appendicular weakness, a cerebellar syndrome, mild aphasia, and slight facial asymmetry.

Given the reported history of acute onset 1 h prior to admission, an ischemic etiology was suspected, and thrombolytic therapy was administered. Subsequently, his weakness progressed, and his level of consciousness deteriorated to a Glasgow Coma Scale (GCS) score of 8, necessitating intubation.

Lumbar puncture revealed pleocytosis of approximately 1500/μl with a predominance of polymorphonuclear cells, elevated protein levels, and increased lactate. Positive WNV polymerase chain reaction (PCR) results were obtained from cerebrospinal fluid (CSF), blood, and urine samples collected on various dates. However, the CSF sample tested negative for antibodies against WNV, whereas the serum sample was positive (IgM titer >1:100; IgG titer >1:100).

After a five-day course of 1 g methylprednisolone and three days of intravenous immunoglobulin (IVIG) treatment (2 g/kg), the patient's symptoms subsided. Twelve days after initial admission to the ER, the patient's vigilance improved, and he was successfully extubated after 10 days. Fourteen months after the infection, the patient continues to suffer from a marked decrease in physical ability.

An 86-year-old active male patient developed sudden fever, diarrhea, a maculopapular rash and confusion. Empirical treatment with ampicillin/sulbactam was initiated, assuming a bacterial etiology. However, due to worsening clinical status and neurological decline, the patient was transferred to our clinic.

On admission, he was disoriented and exhibited intermittent, arrhythmic myoclonus in the left arm, moderate appendicular weakness, and dysarthria. Cerebrospinal fluid (CSF) analysis revealed a markedly increased cell count of 800/μl with a predominance of polymorphonuclear cells, elevated protein levels, and increased lactate. Positive WNV polymerase chain reaction (PCR) results were obtained from CSF, blood and urine samples. Serum analysis revealed IgM and IgG antibodies against WNV (IgM titer >1:100; IgG titer >1:100), although these antibodies were not detected in the CSF. Treatment with 60 mg of prednisolone daily was initiated and gradually tapered over six weeks.

On day 6 of hospitalization, the patient developed a hemiparesis and MRI revealed acute intracranial hemorrhage in the right thalamus. The patient was subsequently extubated and discharged for early neurological rehabilitation 26 days after admission. At five months post discharge, the patient remains partially dependent on mobility, primarily using a wheelchair and requires assistance due to ongoing cognitive impairment and weakness.

A 69-year-old female teacher was initially admitted to the hospital with fever, gastrointestinal symptoms including vomiting and diarrhea, and progressive weakness following a school trip. Subsequently she experienced neurological deterioration with psychomotor retardation, acute flaccid paralysis, and dysarthria. Cerebrospinal fluid (CSF) analysis revealed pleocytosis with a cell count of 50/μl, predominantly mononuclear cells, increased lactate, and elevated protein levels.

**Fig. 1:**
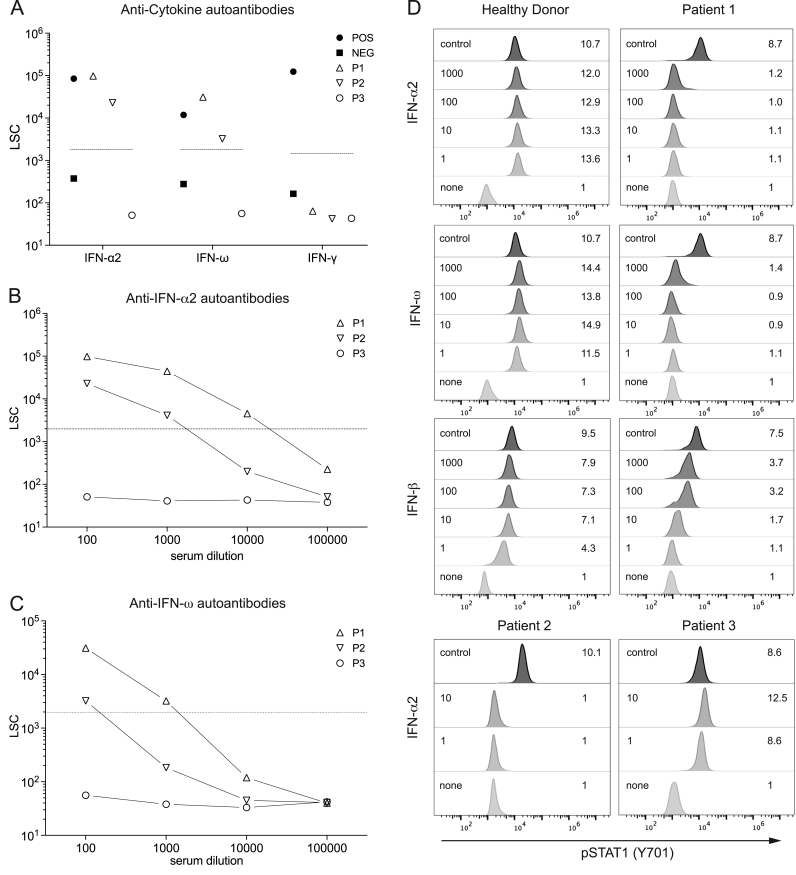
**Neutralizing auto-Abs against IFN-α2 and IFN-ω in patients with WNV encephalitis.** (**A**) Detection of IgG auto-Abs against IFN-α2, IFN-ω and IFN-γ in sera (1:100 dilution) from patients infected with WNV. Sera from patients with or without auto-Abs against IFN-α2, IFN-ω and IFN-γ served as positive and negative controls, respectively. Samples whose test results exceeded assay-specific cut-off values (dotted lines) were considered “positive” for the respective auto-Ab. Detection of auto-Abs against (**B**) IFN-α2 and (**C**) IFN-ω in serially diluted patient sera. (**D**) FACS histograms show intracellular STAT1 phosphorylation (pSTAT1) in whole-blood monocytes from a healthy control and patients 1 to 3 stimulated with different concentrations (1, 10, 100 or 1000 ng/ml) of recombinant human IFN-α2 (Merck Sharp & Dohme), of recombinant human IFN- ω (Boehringer Ingelheim) or of recombinant human IFN- β (Bayer Vital GmbH). As a positive control, cells were stimulated with 10 ng/ml IFN- γ (control). The concentration of the respective cytokines and the pSTAT1 stimulation index (ratio between mean fluorescence intensity (MFI) of the stimulated and unstimulated samples) are denoted on the left and right side of each histogram, respectively.

PCR testing for West Nile virus (WNV) returned positive results in both the urine samples and the EDTA-plasma samples. Serum analysis revealed IgM and IgG antibodies against WNV (IgM titer >1:100; IgG titer >1:100), although both CSF antibody testing and PCR testing for WNV in CSF were negative. Notably, no neutralizing antibodies against interferon-α and/or interferon-ω were detected (Fig. 1A–C).

Therapy was initiated with 500 mg of prednisolone daily for five days, along with intravenous immunoglobulin (IVIG) at a dose of 2 g/kg (totaling 160 g over four days) leading to improvement, four months after the onset of symptoms, she continues to suffer from muscle weakness in her legs, resulting in gait instability and reliance on a rollator walker.

### 3.2 Virological data

Positive WNV polymerase chain reaction (PCR) results were obtained in all three patients from various specimen types. Complete genome sequencing revealed a lineage 2 sequence with high similarity to WNV isolates from other autochthonous human cases, birds, and mosquitoes in Germany (Fig. 1). In the national reporting system of the Robert Koch Institute only 17 and 45 case of human WNV infections were reported in 2023 and 2024 (Robert-Koch-Institut. Survstat).

Patients were primarily over the age of 55 and resided in eastern Germany, with no significant sex differences reported. According to monitoring activities by the Friedrich Loeffler Institute (FLI), the German Federal Authority for Animal Health, WNV subtype 2 has been detected in birds and horses in Germany since 2018 (3). Although eastern Germany is considered the main area of WNV circulation, the virus has been increasingly detected in animals in other regions. Additionally, WNV has been detected in the local mosquito species complex Culex pipiens, a known vector.

### 3.3 Autoantibody data

**Fig. 2:**
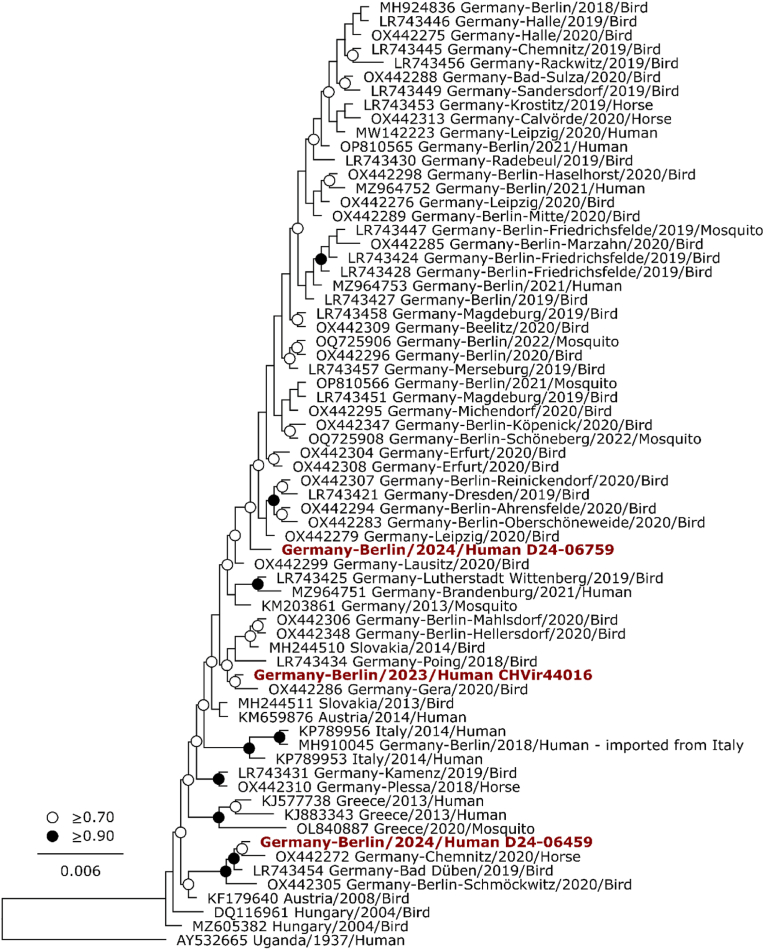
Phylogenetic relationship of the partial coding region of West Nile virus lineage 2 strains. For patient Three only partial genome information could be obtained. The genome regions analyzed comprise the genome positions 1168–1784 and 3601–4886 relative to the reference sequence MH924836.1. Reference sequences obtained from GenBank include their accession number, country of origin, sampling year and host in the name. Sequences generated in this study are shown in red. AY532665 was used as an outgroup for rooting of the tree. Black circles at nodes indicate bootstrap support values of >90 % and white circles >70 %. Phylogenetic analysis was conducted using the FastTree v2.1.11 Plugin in Geneious Prime v2022.0.1. (For interpretation of the references to color in this figure legend, the reader is referred to the Web version of this article.)

In our study, we performed immunoassays—including competition assays and STAT1 activation assays—to detect interferon autoantibodies (IFN-AABs) and confirm their functionality (Fig. 2). Neutralizing antibodies against IFN-α and/or IFN-ω were detected in Patients One and Two but not in Patient Three. FACS analysis confirmed their neutralizing effect by demonstrating reduced pSTAT1 levels in monocytes after stimulation with recombinant IFN-α and/or IFN-ω at concentrations up to 1000 ng/ml. Additionally, Patient One also had neutralizing antibodies against IFN-β.

## 4 Discussion

We present three cases of life-threatening autochthonous WNND, with encephalitis and flaccid paralysis occurring during the summer of 2023–2024. Despite their advanced age, all patients were previously active and free of significant comorbidities; however, they developed severe symptoms. Neuroinvasive disease is a rare clinical manifestation of WNV infection, occurring in fewer than 1 % of cases. Notably, all our patients experienced gastrointestinal symptoms and fever followed by a rash at the onset of infection, a feature that is relatively uncommon in other types of encephalitis. Typically, WNND presents as one of three clinical syndromes: meningitis, encephalitis, or flaccid paralysis (Kramer et al., 2007). Features such as muscle weakness, diminished reflexes, and flaccid paralysis are unusual in other forms of encephalitis.

Patient One initially exhibited a cerebellar syndrome characterized by weakness, ataxia, nystagmus, dysarthria, aphasia, and mild facial asymmetries which led to the initial diagnosis of vertebrobasilar infarction and the initiation of thrombolytic therapy. Large case studies of WNND have reported a high incidence of abnormal cranial nerve function resembling stroke symptoms (Hart et al., 2014). Furthermore, Patient One developed flaccid paresis with electroneurographic findings consistent with mixed axonal-demyelinating polyneuropathy and a predominantly motor deficit—findings typical of WNND. Patient Two presented with substantial cognitive difficulties and reduced arousal, consistent with encephalitis, in addition to intermittent myoclonus and hypokinetic–rigid symptoms reminiscent of Parkinson's disease, which have also been described in WNND. In contrast, Patient Three's symptoms were less severe; she exhibited fewer alterations in consciousness and milder cognitive impairment. Her primary neurological manifestation was flaccid paralysis resembling Guillain–Barré syndrome (GBS), which has been described before (Kramer et al., 2007).

All patients met the United States Centers for Disease Control and Prevention (CDC) laboratory diagnostic criteria for WNV infection (Prevention CfDCa). Notably, none of the patients had detectable IgM antibodies in the CSF despite positive serum findings. Given that IgM antibodies do not cross the blood–brain barrier and are typically indicative of WNND, their absence in the CSF is striking, even in the context of positive PCR results. Among patients with WNND, the sensitivity of nucleic acid testing via PCR is approximately 55 % for CSF and 15 % for blood (Nash et al., 2001). In repeated lumbar punctures, the PCR results became negative, yet IgM antibodies remained undetectable in the CSF. Interestingly, PCR tests of urine and serum samples remained positive even 10 days after diagnosis, which is unusual since viremia typically occurs at the beginning of the disease. This persistent positivity may indicate an impaired immune response and reduced antibody production, possibly explaining the absence of IgM antibodies in the CSF.

In Patient Three, neither IgM antibodies nor a positive PCR were detected in the CSF; however, the diagnosis was confirmed by positive PCR results in blood and urine and by the presence of serum IgM antibodies. Although CSF PCR has low sensitivity and the virus may have already been cleared, IgM antibodies generally remain detectable for several months. The absence of detectable antibodies in Patient Three's CSF might be attributable to long-term immunosuppressive therapy with methotrexate. Furthermore, because she did not have IFN autoantibodies her immune system exhibited a stronger antiviral response.

Male sex and advanced age are recognized risk factors for WNND, with a markedly higher prevalence in individuals over the age of 50 Sutinen et al., 2022. A large study of WNND patients in the EU and USA revealed that approximately 40 % of patients with encephalitis carried autoantibodies neutralizing interferon (IFN)-α and/or IFN-ω, whereas virtually no asymptomatic WNV-infected individuals presented such antibodies (Gervais et al., 2023). The presence of type I IFN antibodies may partly explain the severe clinical course in Patients One and Two, highlighting the need for autoantibody screening in older patients suspected of WNV infection. Our findings align with recent reports that indicate a higher prevalence of type I IFN autoantibodies in older individuals and males. Although the mechanisms underlying their development, including the observed gender bias and age-related increase, remain unclear, preexisting type I IFN autoantibodies appear to heighten the risk of severe viral infections, including those caused by SARS-CoV-2, influenza, West Nile, Powassan, Usutu, and Ross River viruses (Bastard et al., 2021; Zhang et al., 2022; Gervais et al., 2024).

Although the mechanism of WNV neuroinvasion remains a subject of ongoing debate, preclinical models have demonstrated that blocking interferon signaling leads to increased permeability of the blood–brain barrier (BBB) and the intestinal barrier (Lin et al., 2023).

The results of the WNV sequences of our patients together with the absence of a travel history highly suggest autochthonous infection with a local lineage. All patients reported engaging in outdoor activities in rural areas where infection likely occurred.

Considering that only approximately 1 % manifest as WNND and revising the epidemiological data described above, substantial underreporting is likely. The true incidence of WNF in Germany may be highly underestimated.

Ecological changes, similar to those observed in the United States following the first WNV epidemic in New York in 1999, suggest that the number of infections in Germany is likely to increase in the coming years (Nash et al., 2001).

All patients received treatment with high-dose corticosteroids and two patients additionally received intravenous immunoglobulins. If clinical improvement was attributable to the treatment or occurred spontaneously remains unclear. Currently, there is no established role for specific antiviral agents, although some studies have reported potential benefits from the use of corticosteroids and intravenous immunoglobulins (Pyrgos and Younus, 2004; Srivastava et al., 2015).

All of our patients experienced long-term physical and cognitive dysfunction, resulting in a substantial loss in quality of life. In longitudinal cohort studies, only a minority of patients achieve full recovery after WNND, with many reporting persistent symptoms such as muscle weakness, impaired concentration, and confusion (Hart et al., 2014).

## 5 Conclusion

In summary, we describe three life-threatening cases of autochthonous West Nile virus infection in Berlin that presented common general symptoms, but distinct neurological manifestations associated with differences in the immune response and the presence of viruses in the central nervous system. Our findings demonstrate that the presence of type I interferon autoantibodies is associated with increased severity of WNND. Given that the prevalence of these autoantibodies increases with age, our results indicate that elderly individuals are at particularly high risk for severe WNND, which is associated with substantial mortality and morbidity. We further hypothesize that the true prevalence of WNV in Germany is likely underestimated, emphasizing the need for increased clinical awareness and enhanced reporting by veterinary and public health organizations.

## CRediT authorship contribution statement

**Jan Braune:** Writing – original draft, Methodology, Investigation, Conceptualization. **Lorenz Pechstein:** Writing – original draft, Investigation. **Christian Meisel:** Writing – review & editing, Investigation. **Tim Meyer:** Investigation. **Julia Melchert:** Investigation. **Victor Max Corman:** Investigation. **Christiana Franke:** Writing – review & editing, Conceptualization. **Thomas Schneider:** Writing – review & editing, Conceptualization.

## Patient consent

Written informed consent was obtained from the patient for publication of this Case report and any accompanying images.

The study has been carried out in accordance with The Code of Ethics of the World Medical Association (Declaration of Helsinki) for experiments involving humans.

## Availability of data and materials

All data and materials are available upon request.

## Funding

This research did not receive any specific funding. Jan Braune is funded by the Berlin Institute of Health (BIH) Clinician Scientist program.

## Declaration of competing interest

The authors declare that they have no known competing financial interests or personal relationships that could have appeared to influence the work reported in this paper.

## Data Availability

Data will be made available on request.

## Glossary

AAB(s): Autoantibody(s)
BBB: Blood–Brain Barrier
CDC: Centers for Disease Control and Prevention
CD: Cluster of Differentiation (e.g., CD16, CD56, CD19, CD3, CD14, CD4, CD45)
CSF: Cerebrospinal Fluid
ECLIA: Electrochemiluminescence Immunoassay
EDTA: Ethylenediaminetetraacetic Acid
ELISA: Enzyme-Linked Immunosorbent Assay
FLI: Friedrich Loeffler Institute
FACS: Fluorescence-Activated Cell Sorting
GBS: Guillain–Barré Syndrome
GCS: Glasgow Coma Scale
IFN: Interferon
IFN-AAB(s): Interferon Autoantibody(s)
IFN-α2: Interferon Alpha-2
IFN-ω: Interferon Omega
IFN-β: Interferon Beta
IFN-γ: Interferon Gamma
IgG: Immunoglobulin G
IgM: Immunoglobulin M
IVIG: Intravenous Immunoglobulin
LSC: Light Signal Counts
MFI: Mean Fluorescent Intensity
MTX: Methotrexate
MSD: Meso Scale Discovery
MRI: Magnetic Resonance Imaging
NIHSS: National Institutes of Health Stroke Scale
PCR: Polymerase Chain Reaction
pSTAT1: Phosphorylated Signal Transducer and Activator of Transcription 1
ER: Emergency Room
WNF: West Nile Fever
WNV: West Nile Virus
WNND: West Nile Virus Neuroinvasive Disease
